# Stable multi-infection of splenocytes during SIV infection - the basis for continuous recombination

**DOI:** 10.1186/1742-4690-9-31

**Published:** 2012-04-23

**Authors:** Anke Schultz, Sieghart Sopper, Ulrike Sauermann, Andreas Meyerhans, Rodolphe Suspène

**Affiliations:** 1 Department of Virology, Saarland University, Homburg D-66421, Germany; 2 Department of Hematology and Oncology, Innsbruck Medical University, Innsbruck, Austria; 3 Unit of Infection Models, German Primate Center, Göttingen D-37077, Germany; 4 ICREA Infection Biology Laboratory, Department of Experimental and Health Sciences, University Pompeu Fabra, Barcelona 08003, Spain

**Keywords:** SIV, FISH, Recombination, Provirus copy number

## Abstract

**Background:**

Recombination is an important mechanism in the generation of genetic diversity of the human (HIV) and simian (SIV) immunodeficiency viruses. It requires the co-packaging of divergent RNA genomes into the same retroviral capsid and subsequent template switching during the reverse transcription reaction. By HIV-specific fluorescence *in situ *hybridization (FISH), we have previously shown that the splenocytes from 2 chronically infected patients with Castelman's disease were multi-infected and thus fulfill the *in vivo *requirements to generate genetic diversity by recombination. In order to analyze when multi-infection first occurs during a lentivirus infection and how the distribution of multi-infection evolves during the disease course, we now determined the SIV copy numbers from splenocytes of 11 SIVmac251-infected rhesus macaques cross-sectionally covering the time span of primary infection throughout to end-stage immunodeficiency.

**Results:**

SIV multi-infection of single splenocytes was readily detected in all monkeys and all stages of the infection. Single-infected cells were more frequent than double- or triple- infected cells. There was no strong trend linking the copy number distribution to plasma viral load, disease stage, or CD4 cell counts.

**Conclusions:**

SIV multi-infection of single cells is already established during the primary infection phase thus enabling recombination to affect viral evolution *in vivo *throughout the disease course.

## Background

The human (HIV) and simian (SIV) immunodeficiency viruses exhibit phenomenal genetic diversity. This diversity is generated by 3 different mechanisms, (i) error-prone replication by the reverse transcriptase that occurs without proofreading [1], (ii) hypermutation by host mutators of the family of cytidine deaminases [2-6] and (iii) recombination between the viral RNA genomes by template switching [7]. As a result of these processes, a population of virus variants or quasispecies is established within an infected host that is subsequently shaped by processes like fitness competition between variants, immune-mediated activation of infected cells and bottlenecking [8-11].

Due to the structure of the retrovirus particle and the low processivity of the reverse transcriptase, recombination is extraordinary frequent in HIV and SIV [12]. In fact, the number of crossovers during reverse transcription between the two RNA templates that are packaged within one HIV virion has been estimated to be around three or higher per genome [13-16]. Thus any generated single provirus can be expected to have a mosaic structure derived from its two parental viral RNA strands. In comparison, the point mutation rate with approximately 0.25 mutations per genome and replication [17] is at least a factor of 10 lower.

To have an impact on virus evolution, recombination needs to proceed between non-identical RNA strands. The requirements and biological steps for the generation of such virus particles with non-identical genomes have been described. First, multi-infection of single cells occurs via direct and via cell-mediated entry pathways [18]. Second, the existence of multi-infected cells has been directly shown by HIV-specific fluorescence *in situ *hybridization (FISH) on splenocytes of infected patients [19]. Third, transcribed HIV RNA genomes are co-packaged into virions at ratios expected from a random distribution [20]. However, the extent of sequence similarity, especially of the dimerization initiation site, influences co-packaging efficiency and consequently the frequency of observable recombinants [21-23].

While features of the basic steps in the generation of HIV/SIV recombinants have been described in various experimental conditions, it is still unknown when multi-infected single cells first appear *in vivo *during a lentivirus infection and how the distribution of multi-infection evolves during the disease course. To answer these questions, we determined the SIV copy number in splenocytes from SIVmac251-infected rhesus macaques (*Macaca mulatta*) by specific fluorescence in situ hybridization.

## Results and discussion

### Multi-infection of single splenocytes of SIV-infected rhesus macaques in all stages of infection

To determine the SIV copy number of splenocytes from SIVmac251-infected animals by FISH, DNA from SIVmac239 was used as a probe. This was possible due to the high sequence homology between SIVmac239 and SIVmac251 [24]. *In vitro *SIVmac239-infected CEMx174 cells served as positive control to test the sensitivity of the biotin-labelled SIV DNA-probe while un-infected CEMx174 cells as well as spleen cells from an un-infected rhesus macaque were used as negative controls. Only after detecting bright signals in the interphase nuclei of the infected CEMx174 cells with no nonspecific background in the negative samples, the splenocytes of the infected animals were analyzed under the same conditions.

**Figure 1:**
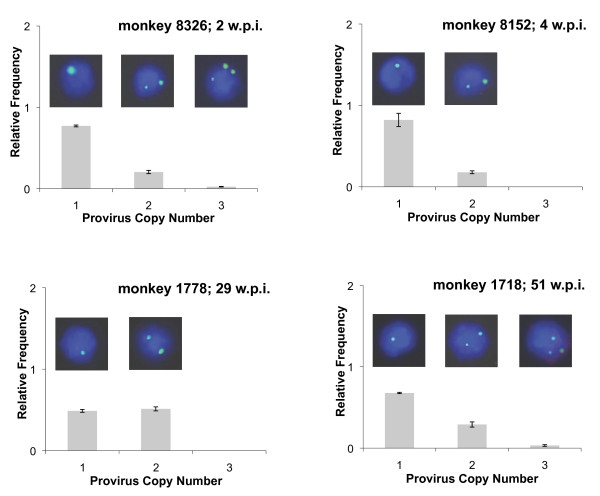
**Distribution of the relative SIV provirus copy number in single spleen cells of SIVmac251-infected rhesus macaques**. The SIV copy number was determined with SIV-specific fluorescence *in situ *hybridization. Results from 4 monkeys are shown. Exact numbers are indicated in Table 1. Pictures from individual nuclei with different SIV copy numbers are given as examples. Green spots correspond to proviral SIV-genomes detected with a biotin-labelled SIVmac239Δenv-probe and a biotin-tyramid signal amplification system. W.p.i. = weeks post infection.

Multi-SIV-infected cells were observed for all animals and throughout the entire observation period from primary infection (2 weeks post infection) to the AIDS state (animals 1740, 1778 and 1718; Figure 1 and Table 1). The relative distribution of provirus copy numbers was similar from the acute to the late stage of infection. With the exception of monkey 1778, cells with 1 provirus copy were more frequent than cells with 2 or 3 copies. Three provirus copies/cell were detected in 2 monkeys at 2 weeks and 51 weeks post infection, respectively. Thus multi-infection of single splenocytes is a general phenomenon that occurs during all stages of an SIV infection.

**Table 1:** Characteristics of SIVmac251-infected rhesus macaques

Monkey	Duration of infection (weeks)	Absolute CD4+ T cells (cell/μl)	Plasma viral load (SIV-RNA/ml)	Number of infected cells counted	Relative SIV copy number distribution		
					
					1 copy	2 copies	3 copies
8310 (I)	0	804	nd	-	-	-	-
8326 (I)	2	1980	nd	83	0.77	0.20	0.02
7783 (C)	2	471	nd	125	0.69	0.31	-
8152 (I)	4	nd	nd	28	0.82	0.18	-
7793 (C)	7	471	nd	122	0.80	0.20	-
8179 (C)	12	252	7.7 × 10^3^	52	0.73	0.27	-
1740 (I, A)	13	131	2.3 × 10^8^	77	0.73	0.27	-
7785 (C)	17	910	nd	45	0.67	0.33	-
8159 (I)	18	545	nd	163	0.72	0.28	-
7786 (C)	19	840	nd	111	0.73	0.27	-
1778 (I, A)	29	571	3, 1 × 10^6^	41	0.49	0.51	-
1718, (I, A)	51	54	2.6 × 10^5^	93	0.68	0.29	0.03

Relative SIV copy number distribution including the number of infected cells counted and some characteristics of the SIV infection are given for all monkeys studied. The determined copy numbers are the result of 2 to 4 independent FISH experiments. The standard deviation of individual copy number counts was less than 10%. Around 200 to 300 non-infected control cells were inspected in each FISH experiment. I = Rhesus macaques of Indian origin; C = Rhesus macaques of Chinese origin; A = AIDS state.

To evaluate the efficiency of the FISH assay and the error in our provirus copy number estimates, the technique has to be tested on cells with a defined copy number. For this we used U1 cells that carry 2 HIV provirus copies per genome and performed the assay under identical conditions only using HIV DNA as a probe. This is a valid approach as the base composition and thus the hybridization properties for HIV-1 and SIV are basically identical (HIV-1: A-35,3%, T-22,1%, G-24,4%, C-18,2% and SIV239: A-33,6%, T-22,7%, G-24,7%, C-18,8%). Approximately 10% of U1 cells showed only one copy. Thus it would appear that we underestimated the real values by ~10%. Within the same experiments, we very rarely detected 3 proviruses (< 0.5% of cells). Likewise, using either HIV DNA or SIV DNA as probes, non-infected monkey splenocytes rarely gave a positive signal (< 0.5%) suggesting that the number of false positive events is very small. Extrapolating these findings to the SIV FISH experiments with splenocytes from infected monkeys, the real SIV proviral copy number may be around 10% higher than indicated.

While the sample size of this present study is low and thus has limited statistical power, there was no strong trend between the SIV plasma viral load, the stage of SIV infection, or the CD4 cell count and the relative copy number distribution. For example, monkeys 8179 and 1740 differed more than 10^4^-fold in plasma virus titres but showed a similar provirus distribution in their splenocytes (Table 1). A similar observation was made previously when studying the HIV copy number distributions in splenocytes of 2 seropositive patients with Castleman disease [19]. Notably, in this latter study, the copy number distribution was higher with the majority of cells harbouring 3 to 4 proviruses. While the reason for this difference is unknown, it may well be related to the lymphoproliferative nature of the disease [25]. Nonetheless, with respect to recombination, any cell with a provirus copy number above 1 is able to generate virions with mixed RNA genomes that in the subsequent infection round will result in recombinant progeny.

How are infected splenocytes with multiple proviruses generated? The lack of a clear trend between the plasma viral load and the copy number distribution of this study and that of Jung *et al*. [19] suggest that cell-cell transmission events rather than circulating virus itself are the likely viral source. Indeed, infections by viruses deposited on Follicular Dendritic cells [26] or transferred from Dendritic cells to T cells in the process of antigen-specific stimulation [27] are highly efficient and may result in the transmission of multiple virions as demonstrated by more recent *ex vivo *studies [28,29].

The early onset of SIV multi-infection provides the fundamental mechanistic requirements for recombination to occur already at primary viremia. Indeed, when macaques were inoculated simultaneously with SIVmac239Δ*vpx *or Δ*vpr *and SIVmac239Δ*nef*, the emergence of wild-type virus was detected in blood in as little as 2 weeks post-inoculation [30,31]. This was due to the large fitness advantage of the recombined wild-type over both deletion mutants. In naturally occurring HIV infections however, only one or very few virus variants are transmitted resulting in a rather homogenous virus population at primary viremia [32,33]. Consequently, any generated recombinant will remain unnoticed unless a fair degree of sequence diversity is generated over time by error prone replication rounds. This scenario is well documented and shows up in the time-structured sequence networks from HIV patients as well as clonal SIV infections of macaques [34-37].

## Conclusions

In summary, SIV multi-infection of single cells within a lymphoid tissue is established during the primary infection phase and observed throughout the entire disease course. As a consequence, recombination events will inevitably occur and contribute to virus evolution.

## Methods

Splenocytes were analyzed from 11 SIVmac251-infected monkeys that had been used in previously published infection studies [38]. Animals were housed at the German Primate Center under standard conditions and experiments were performed according to the German animal protection law which complies with the European Union guidelines on the use of non-human primates for biomedical research (approval 604.42502/08-02.95, Bezirksregierung Braunschweig). The animals had been infected intravenously with 100 MID_50 _(monkey infectious dose infecting 50% of recipients) of a SIVmac251-derived virus stock prepared in rhesus monkey peripheral blood mononuclear cells [39]. They were sacrificed at predetermined time points in the acute, post-acute, and early asymptomatic phase without clinical symptoms of immunodeficiency or euthanized due to early signs of AIDS. The eleven animals cover a time range from 2 weeks to 51 weeks post primary infection and a wide range of peripheral CD4 T cell counts (Table 1). The spleens were removed from anesthetized animals, cut into slices, and put into ice-cold Hank's balanced salt solution containing 3% FCS. Spleen tissue was then forced through a 100 μm-mesh metal sieve with forceps. The dissociated material was left to settle for five minutes, and the single cell suspension in the supernatant was collected by centrifugation at 170 g for 10 minutes at 4°C and washed twice. Cells were counted in a Neubauer chamber and stored in liquid nitrogen. For the SIV-specific FISH, the thawed cells were stimulated for 2 days in the presence of 10 μM AZT. The fixed cell nuclei were then hydridized with a biotin-labelled SIVmac239-specific DNA probe that was subsequently detected with a tyramide signal amplification (TSA) system. The fluorescence signals were analysed with an Olympus AX 70 epifluorescence microscope (Olympus Optical, Hamburg Germany). Pictures were documented with a digital camera Hamamatsu DC C4742-95 (Hamamatsu Photonics Deutschland GmbH, Herrsching am Ammersee, Germany) and an ISIS digital imaging analysis system (Metasystems GmbH, Altlussheim Germany). A detailed description of the experimental procedures is given in the Additional file 1 and Additional file 2.

## Competing interests

The authors declare that they have no competing interests.

## Authors' contributions

RS and AS performed the work. SS and US performed animal studies and collected the samples. RS and AM designed the study and wrote the paper. All authors read and approved the final manuscript.

## Supplementary Material

Additional file 1 **Detailed protocol for the preparation of cells for fluorescence in situ hybridization**. Click here for file

Additional file 2 **Detailed protocol for the SIV-specific fluorescence in situ hybridization**. Click here for file

## References

[B1] Roberts JD Bebenek K Kunkel TA The accuracy of reverse transcriptase from HIV-1 Science 1988 242 1171 1173 10.1126/science.2460925 2460925

[B2] Harris RS Bishop KN Sheehy AM Craig HM Petersen-Mahrt SK Watt IN Neuberger MS Malim MH DNA deamination mediates innate immunity to retroviral infection Cell 2003 113 803 809 10.1016/S0092-8674(03)00423-9 12809610

[B3] Lecossier D Bouchonnet F Clavel F Hance AJ Hypermutation of HIV-1 DNA in the absence of the Vif protein Science 2003 300 1112 10.1126/science.1083338 12750511

[B4] Mangeat B Turelli P Caron G Friedli M Perrin L Trono D Broad antiretroviral defence by human APOBEC3G through lethal editing of nascent reverse transcripts Nature 2003 424 99 103 10.1038/nature01709 12808466

[B5] Mariani R Chen D Schrofelbauer B Navarro F Konig R Bollman B Munk C Nymark-McMahon H Landau NR Species-specific exclusion of APOBEC3G from HIV-1 virions by Vif Cell 2003 114 21 31 10.1016/S0092-8674(03)00515-4 12859895

[B6] Zhang H Yang B Pomerantz RJ Zhang C Arunachalam SC Gao L The cytidine deaminase CEM15 induces hypermutation in newly synthesized HIV-1 DNA Nature 2003 424 94 98 10.1038/nature01707 12808465 PMC1350966

[B7] Coffin JM Structure, replication, and recombination of retrovirus genomes: some unifying hypotheses J Gen Virol 1979 42 1 26 10.1099/0022-1317-42-1-1 215703

[B8] Cheynier R Henrichwark S Hadida F Pelletier E Oksenhendler E Autran B Wain-Hobson S HIV and T cell expansion in splenic white pulps is accompanied by infiltration of HIV-specific cytotoxic T lymphocytes Cell 1994 78 373 387 10.1016/0092-8674(94)90417-0 7914835

[B9] Coffin JM Genetic diversity and evolution of retroviruses Curr Top Microbiol Immunol 1992 176 143 164 10.1007/978-3-642-77011-1_10 1600751

[B10] Gratton S Cheynier R Dumaurier MJ Oksenhendler E Wain-Hobson S Highly restricted spread of HIV-1 and multiply infected cells within splenic germinal centers Proc Natl Acad Sci USA 2000 97 14566 14571 10.1073/pnas.97.26.14566 11121058 PMC18959

[B11] Meyerhans A Cheynier R Albert J Seth M Kwok S Sninsky J Morfeldt-Manson L Asjo B Wain-Hobson S Temporal fluctuations in HIV quasispecies in vivo are not reflected by sequential HIV isolations Cell 1989 58 901 910 10.1016/0092-8674(89)90942-2 2550139

[B12] Chen J Powell D Hu WS High frequency of genetic recombination is a common feature of primate lentivirus replication J Virol 2006 80 9651 9658 10.1128/JVI.00936-06 16973569 PMC1617242

[B13] Jetzt AE Yu H Klarmann GJ Ron Y Preston BD Dougherty JP High rate of recombination throughout the human immunodeficiency virus type 1 genome J Virol 2000 74 1234 1240 10.1128/JVI.74.3.1234-1240.2000 10627533 PMC111457

[B14] Levy DN Aldrovandi GM Kutsch O Shaw GM Dynamics of HIV-1 recombination in its natural target cells Proc Natl Acad Sci USA 2004 101 4204 4209 10.1073/pnas.0306764101 15010526 PMC384719

[B15] Onafuwa A An W Robson ND Telesnitsky A Human immunodeficiency virus type 1 genetic recombination is more frequent than that of Moloney murine leukemia virus despite similar template switching rates J Virol 2003 77 4577 4587 10.1128/JVI.77.8.4577-4587.2003 12663764 PMC152108

[B16] Rhodes TD Nikolaitchik O Chen J Powell D Hu WS Genetic recombination of human immunodeficiency virus type 1 in one round of viral replication: effects of genetic distance, target cells, accessory genes, and lack of high negative interference in crossover events J Virol 2005 79 1666 1677 10.1128/JVI.79.3.1666-1677.2005 15650192 PMC544095

[B17] Mansky LM Temin HM Lower in vivo mutation rate of human immunodeficiency virus type 1 than that predicted from the fidelity of purified reverse transcriptase J Virol 1995 69 5087 5094 7541846 10.1128/jvi.69.8.5087-5094.1995 PMC189326

[B18] Dang Q Chen J Unutmaz D Coffin JM Pathak VK Powell D KewalRamani VN Maldarelli F Hu WS Nonrandom HIV-1 infection and double infection via direct and cell-mediated pathways Proc Natl Acad Sci USA 2004 101 632 637 10.1073/pnas.0307636100 14707263 PMC327199

[B19] Jung A Maier R Vartanian JP Bocharov G Jung V Fischer U Meese E Wain-Hobson S Meyerhans A Multiply infected spleen cells in HIV patients Nature 2002 418 144 10.1038/418144a 12110879

[B20] Chen J Nikolaitchik O Singh J Wright A Bencsics CE Coffin JM Ni N Lockett S Pathak VK Hu WS High efficiency of HIV-1 genomic RNA packaging and heterozygote formation revealed by single virion analysis Proc Natl Acad Sci USA 2009 106 13535 13540 10.1073/pnas.0906822106 19628694 PMC2714765

[B21] Mayr L Powell R Kinge T Nyambi PN Sequence Analysis of the dimerization initiation site of concordant and discordant viral variants superinfecting HIV Type 1 patients AIDS Res Hum Retroviruses 2011 27 1231 1235 10.1089/aid.2011.0010 21453132 PMC3206772

[B22] Nikolaitchik OA Galli A Moore MD Pathak VK Hu WS Multiple barriers to recombination between divergent HIV-1 variants revealed by a dual-marker recombination assay J Mol Biol 2011 407 521 531 10.1016/j.jmb.2011.01.052 21295586 PMC3065980

[B23] Sakuragi J Sakuragi S Ohishi M Shioda T Direct correlation between genome dimerization and recombination efficiency of HIV-1 Microbes Infect 2010 12 1002 1011 10.1016/j.micinf.2010.06.012 20637891

[B24] Naidu YM Kestler HW Li Y Butler CV Silva DP Schmidt DK Troup CD Sehgal PK Sonigo P Daniel MD Characterization of infectious molecular clones of simian immunodeficiency virus (SIVmac) and human immunodeficiency virus type 2: persistent infection of rhesus monkeys with molecularly cloned SIVmac J Virol 1988 62 4691 4696 2846880 10.1128/jvi.62.12.4691-4696.1988 PMC253583

[B25] Oksenhendler E Duarte M Soulier J Cacoub P Welker Y Cadranel J Cazals-Hatem D Autran B Clauvel JP Raphael M Multicentric Castleman's disease in HIV infection: a clinical and pathological study of 20 patients AIDS 1996 10 61 67 10.1097/00002030-199601000-00009 8924253

[B26] Heath SL Tew JG Tew JG Szakal AK Burton GF Follicular dendritic cells and human immunodeficiency virus infectivity Nature 1995 377 740 744 10.1038/377740a0 7477265

[B27] Tsunetsugu-Yokota Y Akagawa K Kimoto H Suzuki K Iwasaki M Yasuda S Hausser G Hultgren C Meyerhans A Takemori T Monocyte-derived cultured dendritic cells are susceptible to human immunodeficiency virus infection and transmit virus to resting T cells in the process of nominal antigen presentation J Virol 1995 69 4544 4547 7769720 10.1128/jvi.69.7.4544-4547.1995 PMC189202

[B28] Chen J Dang Q Unutmaz D Pathak VK Maldarelli F Powell D Hu WS Mechanisms of nonrandom human immunodeficiency virus type 1 infection and double infection: preference in virus entry is important but is not the sole factor J Virol 2005 79 4140 4149 10.1128/JVI.79.7.4140-4149.2005 15767415 PMC1061529

[B29] Del Portillo A Tripodi J Najfeld V Wodarz D Levy DN Chen BK Multiploid inheritance of HIV-1 during cell-to-cell infection J Virol 2011 85 7169 7176 10.1128/JVI.00231-11 21543479 PMC3126592

[B30] Kim EY Busch M Abel K Fritts L Bustamante P Stanton J Lu D Wu S Glowczwskie J Rourke T Retroviral recombination in vivo: viral replication patterns and genetic structure of simian immunodeficiency virus (SIV) populations in rhesus macaques after simultaneous or sequential intravaginal inoculation with SIVmac239Deltavpx/Deltavpr and SIVmac239Deltanef J Virol 2005 79 4886 4895 10.1128/JVI.79.8.4886-4895.2005 15795274 PMC1069535

[B31] Wooley DP Smith RA Czajak S Desrosiers RC Direct demonstration of retroviral recombination in a rhesus monkey J Virol 1997 71 9650 9653 9371629 10.1128/jvi.71.12.9650-9653.1997 PMC230273

[B32] Herbeck JT Rolland M Liu Y McLaughlin S McNevin J Zhao H Wong K Stoddard JN Raugi D Sorensen S Demographic processes affect HIV-1 evolution in primary infection before the onset of selective processes J Virol 2011 85 7523 7534 10.1128/JVI.02697-10 21593162 PMC3147913

[B33] Keele BF Giorgi EE Salazar-Gonzalez JF Decker JM Pham KT Salazar MG Sun C Grayson T Wang S Li H Identification and characterization of transmitted and early founder virus envelopes in primary HIV-1 infection Proc Natl Acad Sci USA 2008 105 7552 7557 10.1073/pnas.0802203105 18490657 PMC2387184

[B34] Kils-Hutten L Cheynier R Wain-Hobson S Meyerhans A Phylogenetic reconstruction of intrapatient evolution of human immunodeficiency virus type 1: predominance of drift and purifying selection J Gen Virol 2001 82 1621 1627 11413373 10.1099/0022-1317-82-7-1621

[B35] Pelletier E Saurin W Cheynier R Letvin NL Wain-Hobson S The tempo and mode of SIV quasispecies development in vivo calls for massive viral replication and clearance Virology 1995 208 644 652 10.1006/viro.1995.1195 7747436

[B36] Plikat U Nieselt-Struwe K Meyerhans A Genetic drift can dominate short-term human immunodeficiency virus type 1 nef quasispecies evolution in vivo J Virol 1997 71 4233 4240 9151810 10.1128/jvi.71.6.4233-4240.1997 PMC191638

[B37] Wain-Hobson S Renoux-Elbe C Vartanian JP Meyerhans A Network analysis of human and simian immunodeficiency virus sequence sets reveals massive recombination resulting in shorter pathways J Gen Virol 2003 84 885 895 10.1099/vir.0.18894-0 12655089

[B38] Sopper S Sauer U Hemm S Demuth M Muller J Stahl-Hennig C Hunsmann G ter Meulen V Dorries R Protective role of the virus-specific immune response for development of severe neurologic signs in simian immunodeficiency virus-infected macaques J Virol 1998 72 9940 9947 9811731 10.1128/jvi.72.12.9940-9947.1998 PMC110507

[B39] Stahl-Hennig C Voss G Dittmer U Coulibaly C Petry H Makoschey B Cranage MP Aubertin AM Luke W Hunsmann G Protection of monkeys by a split vaccine against SIVmac depends upon biological properties of the challenge virus AIDS 1993 7 787 795 10.1097/00002030-199306000-00005 8363756

