# Supplementary material for: Stable multi-infection of splenocytes during SIV infection - the basis for continuous recombination
Source: Retrovirology. 2012 Apr 23;9:31. doi: 10.1186/1742-4690-9-31 (PMC3395872; doi:10.1186/1742-4690-9-31)
Supplement: Additional file 1 — Detailed protocol for the preparation of cells for fluorescence in situ hybridization. [file 1742-4690-9-31-S1.DOCx]

## Additional file 1

#### Title: Detailed protocol for the preparation of cells for fluorescence in situ hybridization

Description: Frozen spleen cells from SIV-infected and control monkeys were thawed and 1 x 10^6^ cells/ml were stimulated with 2.5 µg/ml phythaemagglutinin (PHA) (Difko, Detroit, MI, USA) in the presence of 10 µM azidothymidine (AZT) (Sigma-Aldrich, St. Louis, MO, USA) to prevent the virus spread in the cell culture. After two days of culture in RPMI 1640 (Lonza Walkersville Inc, Walkersville, MD, USA) with 10% fetal calf serum (Life Technologies, Paisley, UK), 1% penicillin/streptomycin (Biochrom AG, Berlin, Germany) and IL-2 (100 U/ml; Novartis, Basel, Switzerland), the cell nuclei were prepared via incubation with 0.1 M KCl for 30 min at 37°C, fixed with methanol/acetic acid 3:1 at 4°C and stored at -20°C. As a positive control for the SIVmac-specific FISH, the human T- and B-lymphoblastoid cell line CEMx174 was infected with SIVmac239 with a multiplicity of infection (MOI) of 10. Cells were cultured for 4 days in RPMI 1640 with 10% fetal calf serum and 1% penicillin/streptomycin at 37°C in a humidified atmosphere containing 5% CO_2_. After nuclei preparation with 0.075 M KCl for 6 min, the cells were fixed in methanol/acetic acid 3:1 at 4°C and stored at -20°C.
