# Supplementary material for: Stable multi-infection of splenocytes during SIV infection - the basis for continuous recombination
Source: Retrovirology. 2012 Apr 23;9:31. doi: 10.1186/1742-4690-9-31 (PMC3395872; doi:10.1186/1742-4690-9-31)
Supplement: Additional file 2 — Detailed protocol for the SIV-specific fluorescence in situ hybridization. [file 1742-4690-9-31-S2.DOCx]

## Additional file 2

#### Title: Detailed protocol for the SIV-specific fluorescence in situ hybridization

Description: The SIVmac-specific FISH was performed according to our previous HIV protocol with slight modifications (Jung et al., 2002). Prior hybridization the methanol/acetic acid fixed nuclei were dropped onto slides, dehydrated through 70%, 80% and 90% ethanol (3 min per step) and air-dried. The slides were treated with 25 µg/ml RNase (Roche, Basel, Switzerland) in 2 x standard sodium citrate (SSC) at 37°C for 1 h and then washed 4 x with 2 x SSC. After digestion with 30 µg/ml pepsin (Sigma-Aldrich, St. Louis MO, USA) for 6 min at 37°C and a rinse for 5 min with phosphate-buffered saline (PBS) the nuclei were post-fixed with 2.6% formaldehyde, 0.05 M MgCl_2_ in PBS for 5 min. After a washing step for 5 min in PBS, the nuclei were dehydrated in an ethanol series (70%, 80% and 90%) 3 min each step and air-dried. As probe a biotin-labelled double-stranded DNA probe with a length between 100 and 600 bp was generated via nick translation of the plasmid pSIVmac239Δ*env* according to manufactures protocol (Roche). The denaturation of target DNA and probe DNA occurred consecutively. First the nuclei on the slides were incubated in 0.2 x SSC, 70% formamide for 7 min at 75°C, dehydrated in an ethanol series (70%, 80%, and 100%) each step for 3 min at -20 °C and air-dried. Secondly the hybridization mixture containing the probe DNA (100 ng), 5 µg human Cot-DNA, 5 µg herring sperm-DNA (Roche), 50% formamide, 10% dextran sulphate and 0.05 M sodium phosphate buffer was heated for 7 min at 75°C, followed by 2 min on ice and 12 min at 37°C. Then the hybridization was performed at 37°C overnight in a humid chamber. To remove the unspecific bound probe the slides were rinsed 3 x 5 min at 45°C in 50% formamide, 2 x SSC and additional 3 x 4 min at 45°C in 0.1x SSC. Finally the hybridized probe was detected with a tyramide signal amplification (TSA) system (Perkin-Elmer, Waltham, Massachusetts, USA) according to the manufactures protocol. For blocking slides were placed for 30 min at 37 °C in TNB buffer (0.1 M Tris/HCl, 0.15 M NaCl and 0.5 % blocking reagent). The biotin-labelled DNA probe was detected with Streptavidin-conjugated horseradish peroxidase in 1:75 dilution in TNB buffer for 30 min at 37 °C. For signal amplification the slides were incubated with biotinyl tyramide diluted 1:30 in amplification reagent for 20 min at 37 °C. In the last step Streptavidin Fluorescein (Dianova, Hamburg, Germany) was diluted 1:400 in TNB, added to the slides and incubated for 30 min at 37 °C. Between each incubation there was a 3 x 5 min washing step at 37°C in TNT (0.1 M Tris/HCl, 0.15 M NaCl, 0.05 % Tween20). In the end the slides were dehydrated with an ethanol series (70%, 80% and 90%), air-dried and the nuclei were counterstained with Vectashield Mounting Medium with DAPI (Vector, Burlingame, CA, USA).
